# Mapping the Nordic Research Landscape for the period 2016-2020: a comprehensive study of research outcomes, collaborations, and impact

**DOI:** 10.12688/f1000research.144036.2

**Published:** 2024-07-29

**Authors:** Aparna Narayan, Bharti Chogtu, Manthan Janodia, Raghu Radhakrishnan, Santhosh K. Venkata

**Affiliations:** 1 Manipal College of Dental Sciences, Manipal Academy of Higher Education, Manipal, Karnataka, 576104, India; 2 Kasturba Medical College, Manipal Academy of Higher Education, Manipal, Karnataka, 576104, India; 3 Manipal College of Pharmaceutical Science, Manipal Academy of Higher Education, Manipal, Karnataka, 576104, India; 4 School of Pharmaceutical Management, IIHMR University, Jaipur, Rajasthan, 302029, India; 5 Manipal Institute of Technology, Manipal Academy of Higher Education, Manipal, Karnataka, 576104, India

**Keywords:** Nordic countries, Bibliometric, Research, Subject profile

## Abstract

**Background:**

This article aims to study the research outcomes of five Nordic countries in terms of research publications, spend on R&D, outcomes and collaborations as these are important parameters to understand research thrust of the countries/regions, in addition to their innovation capability.

**Methods:**

The research outcomes of the Nordic countries in terms of the total number of publications, coauthored publications, publications with corporate collaborators, citations, the Field Weighted Citation Index (FWCI) and publications in different subject areas were retrieved using Scopus and its associate SciVal. The research outcomes were extracted for five years from 2016-2020. In addition, total population, researcher population and research spend of these countries have been obtained from World Bank data available for the year 2021.

**Results:**

The analysis showed that Sweden has the highest population and the highest number of researchers in this region. All countries have the highest number of coauthored publications with the United States, followed by the United Kingdom, except Iceland, which has the second highest number of coauthored publications with Sweden. Denmark, followed by Iceland, stands prominent with reference to having publications with corporate collaborations. Denmark and Sweden have a high percentage of articles in first quartile journals, which is above the average for Nordic countries. Iceland stands at the top with the highest citations, which is depicted by high FWCI. Across subject areas, the Nordic countries have maximum publications in life sciences. Other prominent subject areas include technology and natural sciences.

**Conclusion:**

On analysing the research landscape of Nordic countries, maximum research output is in the field of life sciences and medicine, and most of the coauthored publications of these countries are with the United States. Denmark, with its exemplary research output, excels with maximum papers in top quartile journals and with maximum corporate collaborations and the highest FWCI.

## Introduction

Research is one of the metrics used to evaluate the performance of higher education institutions (HEIs). Investments in research and innovations are indications of a country’s development. Nordic countries have been consistently ranked higher for their research capability and innovation competence. Nordic countries, in Northern Europe and the Northern Atlantic, include Sweden, Denmark, Finland, Iceland and Norway. The countries have been among the top 50 Innovative countries according to Global Innovation Index (GII). The Nordic countries are also ranked among the top 50 in terms of Human Capital and Research, and Infrastructure which demonstrates high research capability of these countries.

Kautto ^1^ provides a detailed description of the features of Nordic countries in terms of good economy and social performance. The article also outlines the developments during the period from 1990 to 2000. Eskildsen et al. ^2^ presents employee characteristics regarding job satisfaction and intrinsic work motivation in Nordic countries, suggesting that the satisfaction level of employees in Nordic countries is high and that Danish workers are the most satisfied. The political system of Nordic countries and its influence on the functioning of Nordic countries was presented by Lane & Ersson. ^3^ Kangas & Palme ^4^ reports on the expansion of the computer-based economy in Nordic countries and how it affects businesses. Martela et al. ^5^ provides a broad representation of the culture, lifestyle, and other aspects of Nordic countries. This paper points out that all five Nordic countries have been in the top 10 countries listed in the “World Happiness Report.” Bibliometric studies show that Nordic countries have a long tradition of epidemiological research. Furu et al. ^6^ presents data on the drug exposure behavior of the five Nordic countries. Lin & Edvinsson ^7^ presents a detailed analysis of the human capital, market capital, process capital, renewal capital, financial capital, and other indicators of 40 countries from 1994 to 2005. The analysis in this paper identifies Sweden as having the highest overall intellectual capital, followed by Finland, Switzerland, Denmark, the USA, Norway, and Iceland. These results show that all five Nordic countries are among the top 10 for intellectual capital. Along with the regular professional education offered by HEIs, vocational training plays a vital role. Michelsen et al. ^8^ presents a detailed report on vocational training in Nordic countries. Tossebro et al. ^9^ reports on the reforms carried out by countries during the 1990s to improve conditions for people with intellectual disabilities in Nordic countries. The author presents the trends from a political science perspective and in the context of public management. Loof et al. ^10^ presents survey data from the Organization for Economic Cooperation and Development for Nordic countries such as Finland, Norway, and Sweden at the firm level on two key areas of innovation behavior and innovation contribution to economic growth, concentrating on links between productivity and innovation. Glanzel ^11^ surveys publication activity and citation impact in Scandinavian countries from 1980 to 1997 and reports that Nordic countries have strong co-authorship with Western European and North American countries.

Clarke et al. ^12^ reports the bibliometric overview of public health research in Europe as a part of a collaborative study under “Strengthening Public Health Research in Europe” using the publications indexed in the Science Citation Index and the Social Science Citation Index for the period from 1995 to 2004. Publication output was analysed in terms of population, gross domestic product, and the burden of disease. This research found that Eastern and Southern European countries have lower numbers than Northern European countries. Melin et al. ^13^ identifies the collaborative pattern of all Nordic countries, the UK, and the Netherlands in work published in science citation indexed journals. The behavior of HEIs in these regions is shown to be more or less similar in the proportion of international and national collaborative publications, with more than 50% of the publications having international coauthors.

Gjersvik et al. ^14^ used PubMed to analyse the publication history of Nordic countries such as Sweden, Denmark, Finland, and Norway in dermatology from 1989 to 2008. Articles were analysed based on the country of the first author’s address. Sweden had the highest number of publications, followed by Denmark, Finland, and Norway. Schneider ^15^ presents an analysis of publication details from the NORIA network of Nordic countries, extracting essential information and reporting on the perspectives on reaching excellence. Sandnes ^16^ presents a bibliometric study on human–computer interaction in Nordic-Baltic countries. The results show that Finland, Sweden, and Denmark dominate the region and that collaborative activities are prominent in top-tier conferences compared to entry-level conferences. However, journals are more effective than conferences in generating citations. Features of the International Science Index (ISI) proceedings database, such as information flow, relative attractions, mobility, and country affinity, are analysed by Glanzel et al., ^17^ along with information on conference locations in the sciences, social sciences, and humanities. Koskinen et al. ^18^ is a bibliometric evaluation of scientific work published in Finland from 1996 to 2005, arising from the use of schizophrenia as a keyword in the Web of Science (WoS) database index. More than 40% of the publications involved international collaboration. Tarkowski ^19^ presents a detailed analysis of publication outcomes in environmental health research across Europe over 10 years, showing that Switzerland produces the highest number of publications in Europe. Rabow ^20^ uses bibliometric data to present a detailed feedback model for the universities and colleges of Nordic countries. Merigo et al. ^21^ analyses research in fuzzy sciences in Nordic countries from a quantitative perspective using the publication index of the WoS database. The analysis provides details of key sections, focusing on journal relevance, authors, institutions, and countries. Hanvold et al. ^22^ presents a systematic review of 12,528 articles published from 1994 to 2014 in major indexing agencies on risk factors for occupational accidents and illnesses among young workers in Nordic countries. Widfeldt ^23^ reports a geopolitical study and its influence on Nordic countries such as Denmark, Finland, Norway, and Sweden. A detailed discussion of urban–rural dimensions in the context of Nordic countries is presented by Knudsen, ^24^ which also reports the economic development of Nordic countries after World War II.

HEIs from Nordic countries (Denmark, Norway, Finland, and Sweden) are studied from 1985 to 2010 by Thomsen et al. ^25^ on parameters such as whether there is a social gap in HEIs and whether privileged groups have been able to maintain advantages in higher education. Finland and Norway display a substantial drop in inequality, Denmark shows a modest drop in inequality, and Sweden shows no signs of inequality decreasing as higher education expands. Airey et al. ^26^ presents the influence of English medium instruction in Nordic countries such as Denmark, Finland, Norway, and Sweden. The authors also present the problems faced by faculties and other stakeholders in this form of instruction. Political dialogue and its influence on the lifestyle of HEIs in Nordic countries are reported by Skogerbo et al. ^27^ The effect of supplementary education on HEIs is reported by Christensen. ^28^ Tikkanen ^29^ presents the effect of the technology-intensive work culture of people in Nordic countries such as Denmark, Finland, Norway, and Sweden. Baccini et al. ^30^ reports quantitative indicators measuring the performance of Italian researchers using their citations from 2000 to 2016, describing self-citation and cross-citation strategies used as indicators in science policy contents. Countrywide bibliometric analysis of publications and average citation impact in oncology for articles published in the WoS are reported by López-Illescas et al., ^31^ indicating that countries with larger publication numbers showed a decline in average citation numbers.

Zacca-González et al. ^32^ reports a bibliometric study of publications in public health, environmental, and occupational health for the period from 1996 to 2011 in Scopus-indexed journals. This research presents a detailed analysis of publications from Latin American quantitative (number of publications), qualitative (citations), and collaborative research. Khiste & Paithankar ^33^ reports a bibliometric study of publications in sciences, social sciences, arts, and humanities published from 2008 to 2016 and indexed in Scopus. Country-level analysis of collaborative publication data using Scopus-indexed data from 2003 to 2016 is reported in Tibaná-Herrera et al. ^34^ Shehatta & Al-Rubaish ^35^ reports the details of publications with country-related self-citation and their impact on total citations and average citations, percentage citations, and productive research from 1996 to 2015 for publications indexed in the Scopus database. Self-citation had a strong impact on a country’s scholarly performance, and the removal of self-citation would show the real impact of a country’s research. A graphical representation of journal and country ranking conducted in SCImago is described by Hassan-Montero ^36^ in a map showing the relational matrix of citation, coauthored citation, and other bibliometrics from Scopus-indexed journals. Archambault et al. ^37^ reports a comparison of the Scopus and WoS databases. Lin et al. ^38^ reports research output on four clusters, namely, countries, research subjects, patterns of research, and publication numbers in WoS, along with the subject-wise clusters across countries. Corrall et al. ^39^ describes bibliometrics and research data management in innovation, network technologies, scholarly communications, and national policy. The identified publications also describe the challenges faced by academic libraries in engaging in data management. Miremadi et al. ^40^ reports a study of innovation and research mechanisms aimed at limiting climate change, presenting a detailed discussion on research on renewable technologies in Nordic countries.

De la Porte et al., ^41^ discussed the political reforms in Nordic countries and how those reforms improve the lives of people in Nordic countries. The paper also discusses a survey of various literature related to political reforms in the nation and how they impact research and education. Karseth et al., ^42^ the authors present various educational reforms/steps proposed by the government to improve the quality of higher education in Nordic countries. Verger ^43^ presents an analysis of how evidence-based policy making and reforms have resulted in an improved higher education landscape and thereby impacted research outcomes. Andersson & Sund ^44^ presents the evaluation of productivity in terms of student achievement in terms of credits, research productivity and staff. Authors provide the details analysis for the above scenarios for the period 2011 to 2016. Sivesind & Karseth, ^45^ presented studying the educational policy of Nordic countries from the viewpoints of researchers from Nordic countries and the US. These publications help to understand the enablers for HEIs to contribute to the research outcome of individuals, institutions and society.

### Research gap

Previous studies have reported bibliometric analysis in different subject areas and its implications. The Nordic countries rank high for their research capability, which is measured using research publications, research grants and funds received, citations, innovation etc. Studies on the innovation practices of Nordic countries have also been reported earlier. However, this research analyses research outcomes of Nordic countries as a region comparing research outcome of the countries in the region regarding publication, citations, collaborations and focus on different subject areas. The present work elucidates the bibliometrics of Nordic countries (Denmark, Sweden, Norway, Finland, and Iceland). Also a detailed analyses of the quantitative and qualitative aspects of the publications is reported in this paper. Such bibliometric analysis can give an insight to the funding agencies about the thrust research areas in these countries.

### Identification of research questions

The aim of this study is to compare the research strengths and research outcomes of the five Nordic countries in terms of researcher population, research spending, scholarly outcomes, and international and corporate collaborations. This research also aims to evaluate the scholarly output of each country based on publications in top quartile journals, publications in different subject areas, and the FWCI of each country in different subject areas.

## Methods

### Data collection

The data was extracted from SciVal database ( www.scival.com) and Scopus databases ( www.scopus.com; RRID:SCR_022559) using the search terms “Denmark”, “Sweden”, “Finland”, “Iceland”, and “Norway” under country filter. The data was extracted from 2016 to 2020. The data were extracted on 1 and 2 November 2021. Generally, five years is an acceptable window for research parameters to be studied, including number of citations that accumulate over a period of time, we selected the previous five year window. The data was collected from Scopus/SciVal for the said window as Scopus/SciVal is the largest scholarly and research database. Publication data was examined for scholarly output (number of publications), collaboration percentage (articles published in collaboration with other national and international institutes, and industry), and subject-wise classification number. Citation-related information for 2016 to 2020 also relates to the period from 2016 to 2021. Other data related to population, researcher population, and research spending as a percentage of countries’ gross domestic product (GDP) were obtained from World Bank data ( www.data.worldbank.org) on 15 November 2021 for the period 2016-2020.

The data regarding researcher numbers as compared to population gives an idea about the percent growth of researcher pool when population increases over a period of time.

The data was analysed using descriptive statistics using MS Excel, which was presented in the form of tables and graphs.

### Inclusion and exclusion criteria

The countries included in the study were Denmark, Sweden, Finland, Iceland, and Norway. The research outcomes included in the study encompassed publications, collaboration with other countries, academic--corporate collaborations, and the Field Weighted Citation Index (FWCI) of the above countries. Research outcomes from 2016 to 2020 were included in the study. Data from the Faroe Islands and Greenland were excluded. Research outcomes before 2016 and after 2020 were also excluded.

### Results of the findings

**Figure 1.**
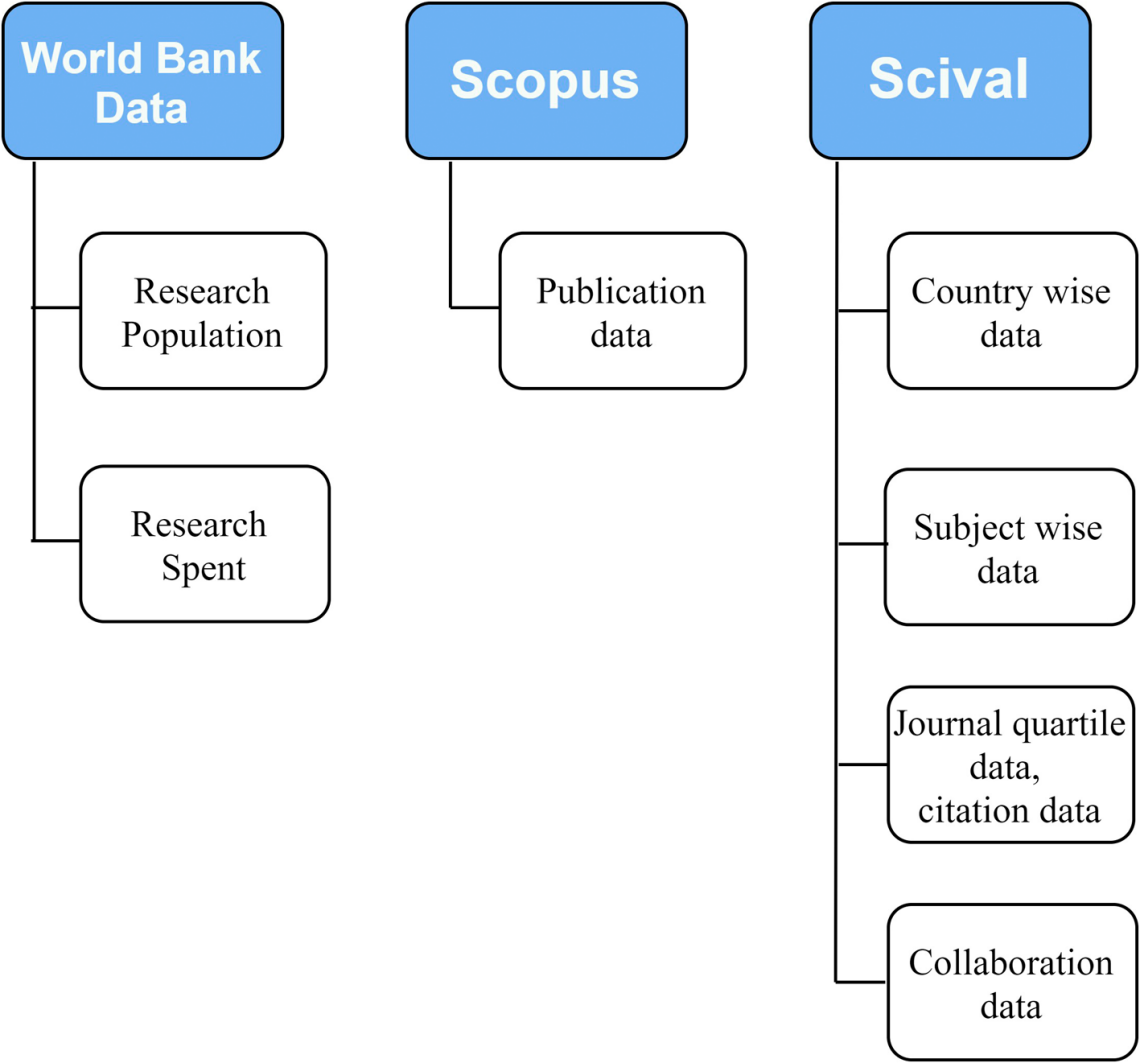
Flow diagram of the data collection process.

The data was retrieved from world bank, Scopus and SciVAL as shown in Figure 1. The consolidated data used for analysis of country wide publication outcome is archived in Ref. 46.

**Figure 2.**
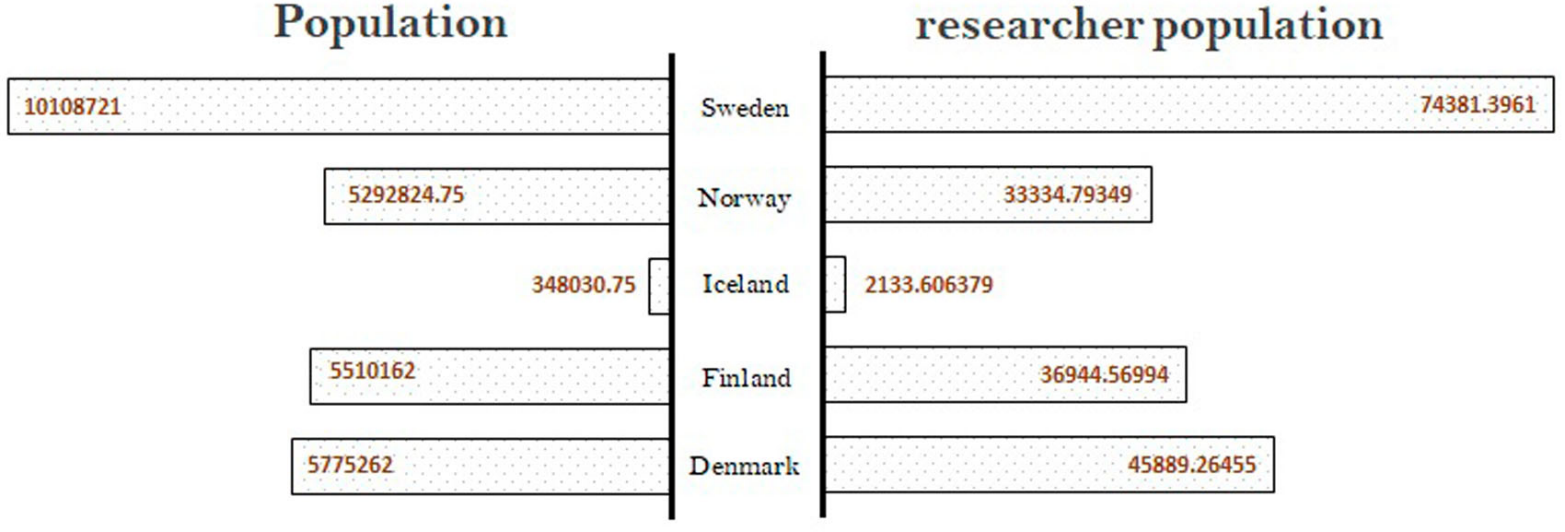
Population of country vs. researcher population in Nordic countries.

**Figure 3.**
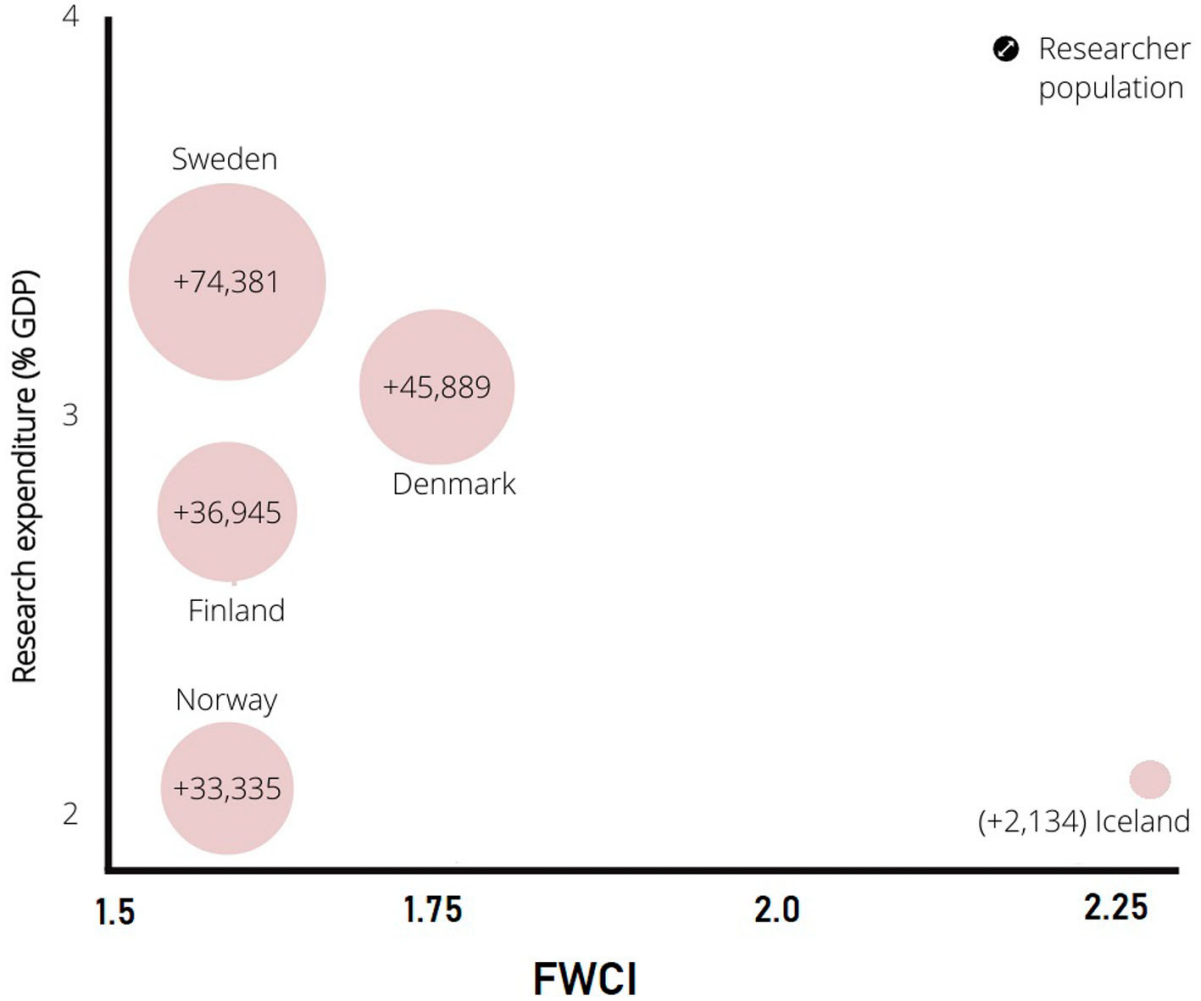
Relation of research spending by percentage GDP to the citation index (Field Weighted Citation Index) vs. the researcher population.

A country’s strength in research, innovation, and technological position can be assessed by its human resource strength. The number of researchers per total population is one of the parameters that provides a reasonable idea of the research strength of a country. In the present research, Figure 2 is a comparative plot indicating the population and researcher population of the Nordic countries. Data related to the population and researcher population were derived from the World Bank database ( https://data.worldbank.org/data extracted on 15 November 2021) for the period from 2016 to 2020. Data show that the Nordic countries have similar percentages of researcher population, Denmark having the highest, followed by Sweden, Finland, Norway, and Iceland. The researcher population is approximately 0.6% to 0.8% of the overall population across all the Nordic countries, with Denmark having a slightly higher number than the other countries. The researcher population is expected to be a parameter influencing the quality of research. Figure 3 presents a comparison of the researcher population of Nordic countries to the qualitative metric of research in these countries (citation-normalized FWCI). The percentage of GDP spent on research is one of the most important parameters driving research worldwide. The present research supplies a graphical representation of the researcher population vs. percentage of GDP spent on research and FWCI. Sweden spends the highest percentage of GDP on research at 3.5%. Sweden also has the highest researcher population among all Nordic countries. Research spending in other Nordic countries, such as Denmark, Finland, Norway, and Iceland, follows Sweden. The researcher population follows the same pattern. The data show that the researcher population directly correlates with the percentage of research spending on GDP in Nordic countries.

Figure 3 provides information on the relation of the percentage of research spending to GDP to the quality of publication in FWCI terms. Field weighted values are globally normalized citation values for a particular subject. The global FWCI is 1. Figure 3 shows that despite lowest percentage of research spend compared to GDP, the quality of publications from Iceland is the highest, which is followed by Denmark. However, Sweden, Finland, and Norway have similar FWCIs. Research spent percentage gives a fair idea of ruling governments commitment to research growth. In general, the percentage spent on research and the researcher population have a direct impact on publication numbers, quality of publications, and citations. In addition, some other parameters impact publication bibliometrics, as discussed in the next sections.

### Collaborative publications

Collaboration is research involving multiple partners contributing to a study. These collaborations may be at the institute level, national level, or international level. Institute-level collaboration involves people from a particular institution working on a common project. National-level collaboration involves people from a particular country joining together to work on a project. International collaboration involves people from different countries. A detailed study was carried out to understand the publication patterns of Nordic countries and their collaborations. Table 1 shows the number of coauthored publications from Denmark with other countries during the 2016–2020 period. The table also indicates the quality of collaboration based on their citation impact on publications. Table 2 provides details of the top collaborative publications from Finland. Similarly, Table 3 shows the outcomes for Iceland. Table 4 shows the output for Norway, and Table 5 shows the output for Sweden. Tables 1 to 5 show that all Nordic countries have the largest number of coauthored publications with the US. The second-largest number of coauthored publications is with the UK. However, Iceland’s second-largest number of coauthored publications is with Sweden. Germany is also one of the most common countries for collaborative research, after the US and the UK. Each of the Nordic countries has different collaborating countries for their fourth collaborative partner. Denmark collaborates with Sweden, the Netherlands, Italy, France, China, Spain, and Australia. Finland collaborates with Sweden, France, Italy, Spain, China, the Netherlands, and the Russian Federation. Iceland’s collaborating countries are Germany, Norway, Denmark, France, the Netherlands, Finland, and Italy. Norway had top collaborations with Sweden, Denmark, Netherlands, France, Italy, Spain, and Canada. Sweden had top collaborations with Italy, France, Netherlands, China, Denmark, Spain, and Norway. It is noteworthy that the top collaborations for Iceland were all European countries. China was the only collaborating Asian country, whereas there were many collaborative publications with Denmark, Finland, and Sweden. Russia’s collaboration with Finland and Norway’s collaboration with Canada were unique. Furthermore, collaboration with Italy and Spain resulted in higher productivity and quality publications among other European countries than did the Nordic countries.

**Table 1.** Top countries with coauthored publications with Denmark.

Denmark		
Country/Region	Number of coauthored publications (2016-2020)	Field-weighted citation impact (2016-2020)
United States	27675	3.31
United Kingdom	23096	3.52
Germany	19977	3.55
Sweden	14300	3.29
Netherlands	12107	4.18
Italy	11708	4.26
France	11342	4.23
China	10305	3.31
Spain	09933	4.41
Australia	09369	4.47

**Table 2.** Top countries with coauthored publications with Finland.

Finland		
Country/Region	Number of coauthored publications (2016-2020)	Field-weighted citation impact (2016-2020)
United States	16830	3.40
United Kingdom	14817	3.62
Germany	13312	3.62
Sweden	12079	3.36
France	08590	4.30
Italy	08452	4.49
Spain	07281	4.76
China	07228	3.68
Netherlands	06912	4.89
Russian Federation	05679	3.82

**Table 3.** Top countries with coauthored publications with Iceland.

Iceland		
Country/Region	Number of coauthored publications (2016-2020)	Field-weighted citation impact (2016-2020)
United States	2271	4.94
Sweden	1830	5.92
United Kingdom	1786	6.40
Germany	1383	7.59
Norway	1379	7.02
Denmark	1349	7.08
France	1033	9.14
Netherlands	0995	9.47
Finland	0943	9.55
Italy	0934	10.1

**Table 4.** Top countries with coauthored publications with Norway.

Norway		
Country/Region	Number of coauthored publications (2016-2020)	Field-weighted citation impact (2016-2020)
United States	19445	3.36
United Kingdom	17695	3.57
Germany	13456	3.80
Sweden	13029	3.33
Denmark	09349	3.59
Netherlands	09088	4.48
France	09047	4.20
Italy	08684	4.53
Spain	07419	4.71
Canada	06479	5.22

**Table 5.** Top countries with coauthored publications with Sweden.

Sweden		
Country/Region	Number of coauthored publications (2016-2020)	Field-weighted citation impact (2016-2020)
United States	39002	3.04
United Kingdom	31680	3.38
Germany	28222	3.13
Italy	16979	3.77
France	16747	3.73
Netherlands	15310	4.09
China	14986	3.14
Denmark	14300	3.29
Spain	13476	4.01
Norway	13029	3.33

**Figure 4.**
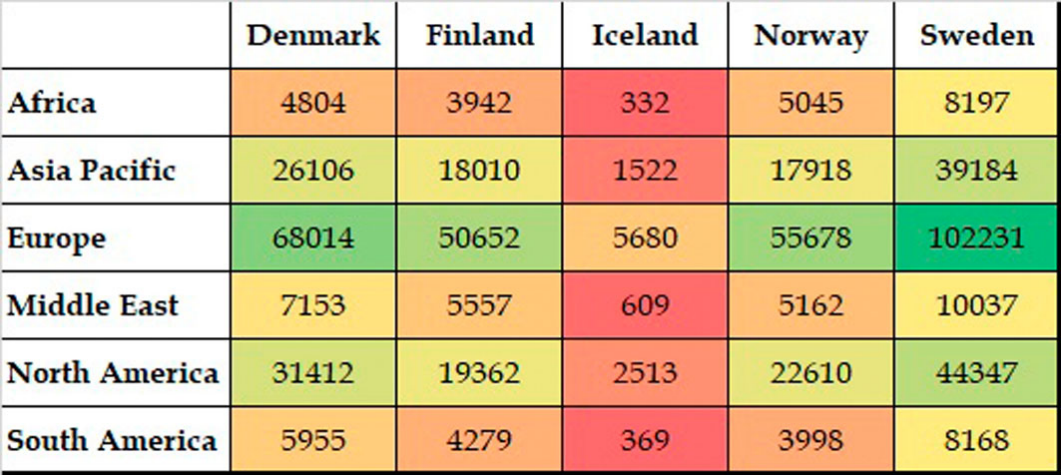
Distribution of coauthored publications across continents.

Figure 4 presents the collective global collaborative publications of Nordic countries. The highest number of collaborative publications was with partners from European countries. This was followed by North America, specifically the USA. The Asia Pacific region was the third top collaborative destination, followed by the Middle Eastern region, South America, and Africa. Collaborative output from the South American and African regions was more or less equal. It is noteworthy that all five Nordic countries presented similar characteristics in terms of collaborations.

Country correlation analysis of co-authored publications in Table 6 shows that the Nordic countries are very strongly connected. Denmark, Sweden, Norway, Finland, and Iceland all have values very close to 1, indicating frequent co-authorship. The highest single correlation is between Denmark and Norway, with 0.99, while the other Nordic pairs are not far behind. By contrast, Greenland, the Faroe Islands, and Svalbard and Jan Mayen indicate weaker but still great cooperation with the Nordic countries. Greenland shows a medium correlation both with Denmark (0.74) and Sweden (0.68). The Faroe Islands also indicate a medium level of collaboration with Denmark and Sweden. However, Svalbard and Jan Mayen have lower correlations with most countries, thus fewer co-authored publications. In general, the data underlines the strong research collaboration within the Nordic region while waning towards the more remote regions.

**Table 6.** Correlation of co-authored publications between different countries.

	Denmark	Sweden	Norway	Finland	Iceland	Greenland	Faroe Islands	Svalbard and Jan Mayen
**Denmark**	1.00	0.96	1.00	0.98	0.99	0.74	0.67	0.60
**Sweden**	0.96	1.00	0.98	1.00	0.99	0.69	0.68	0.40
**Norway**	1.00	0.98	1.00	0.97	1.00	0.53	0.57	0.64
**Finland**	0.98	1.00	0.97	1.00	0.95	0.35	0.41	0.41
**Iceland**	0.99	0.99	1.00	0.95	1.00	0.50	0.59	0.50
**Greenland**	0.74	0.69	0.53	0.35	0.50	1.00	0.98	-0.02
**Faroe Islands**	0.67	0.68	0.57	0.41	0.59	0.98	1.00	0.05
**Svalbard and Jan Mayen**	0.60	0.40	0.64	0.41	0.50	-0.02	0.05	1.00

The correlation analysis in Table 7 reveals that countries with higher scholarly outputs have a significantly higher total number of citations, but this increase in volume does not strongly correlate with higher average citations per publication or field-weighted citation impact. Notably, there is a very strong positive correlation between citations per publication and field-weighted citation impact, suggesting that publications with higher average citations tend to have greater field-adjusted impact. Overall, this indicates that while increasing the number of publications boosts total citations, it is the quality and impact of individual publications that drive higher field-weighted citation impact.

**Table 7.** Correlation between different bibliographic metrics.

	Scholarly Output	Citations	Citation per Publication	Field- Weighted Citation Impact
**Scholarly Output**	1	0.99	-0.19	-0.06
**Citations**	0.99	1	-0.17	-0.04
**Citation per Publication**	-0.19	-0.17	1	0.95
**Field- Weighted Citation Impact**	-0.06	-0.04	0.95	1

### Academia Corporate collaborations

**Figure 5.**
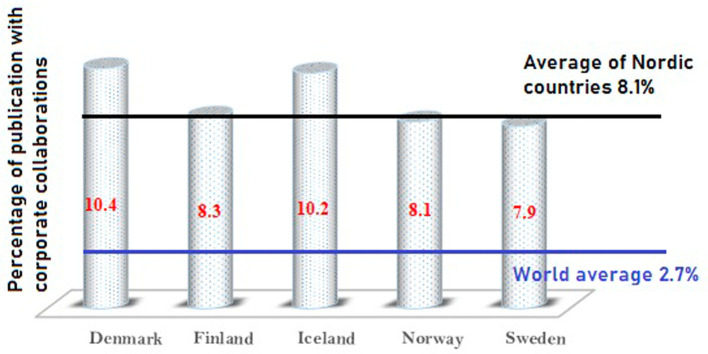
Graph of corporate collaborative publications.

Collaborative publication with industrial coauthors is also one of the important parameters indicating that translational research is happening in an academic or research institution. A country’s corporate academic collaborative publications are reliable indicators of the country’s innovation practices. More academic corporate collaborations would mean more research occurring in the direction of product development, industrial consultancy, and concept to product. An attempt is made in the present research to understand the outcomes of academic corporate collaborations in Nordic countries. Publications from 2016 to 2020 were analysed to identify the percentage of publications with international coauthors, as represented in Figure 5. As Figure 5 shows, Denmark has the highest number of coauthors from industry, approximately 10.4% of its overall publications. Iceland has 10.2%, Finland has 8.3%, Norway has 8.1%, and Sweden has 7.9% of its publications with coauthors from industry. It is important to note that the global average of academic corporate collaborative publications is 2.7%, whereas Nordic countries have an average of 8.1% of their publications with industry collaboration. Nordic countries have an almost three times higher percentage rate than the world average. This indicates that the research carried out in Nordic countries has stronger industry connections when compared to other parts of the world. This is also indicative of a large number of R&D centers in industries in Nordic countries.

### Collaborations among Nordic countries

**Figure 6.**
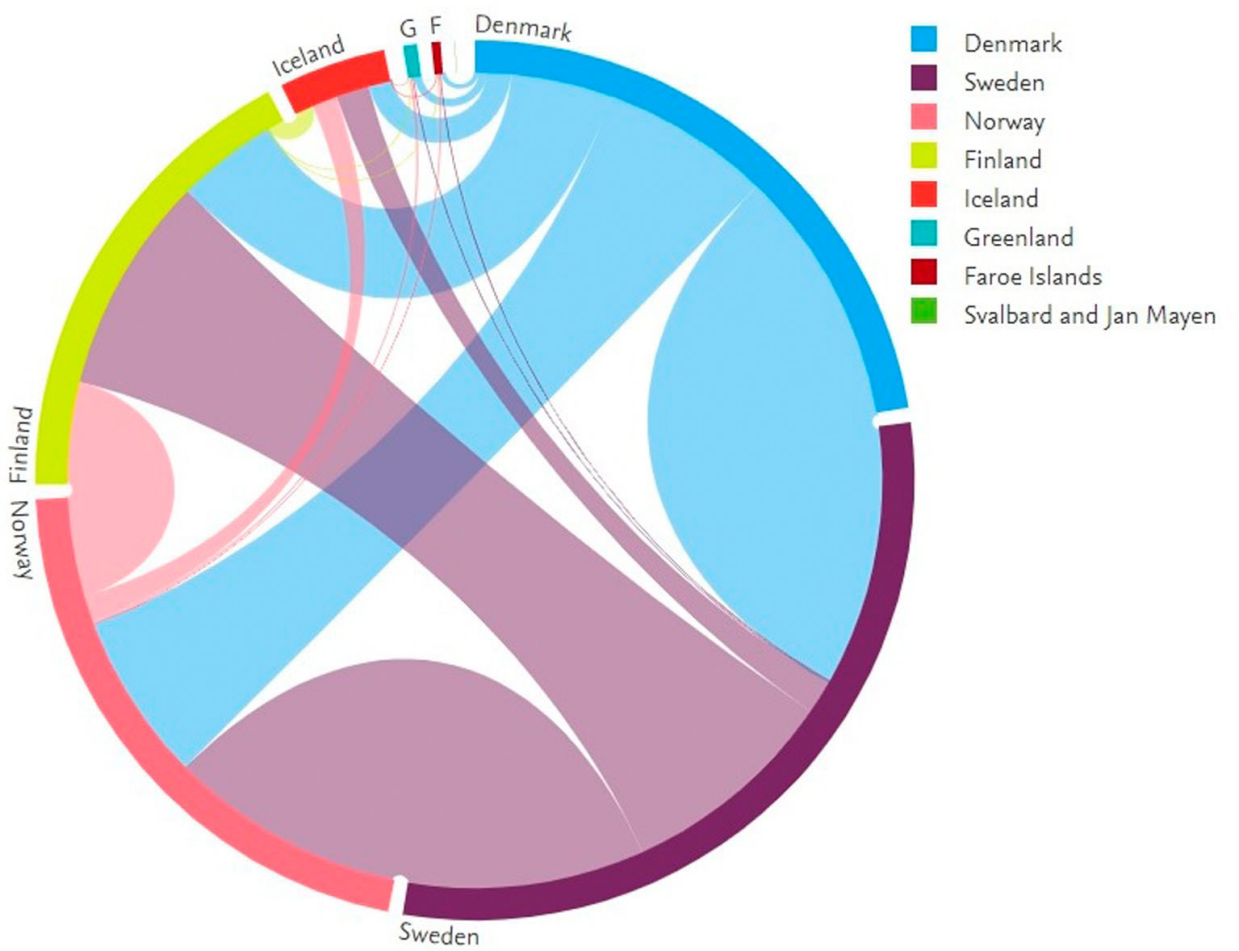
Collaboration among Nordic countries.

**Figure 7.**
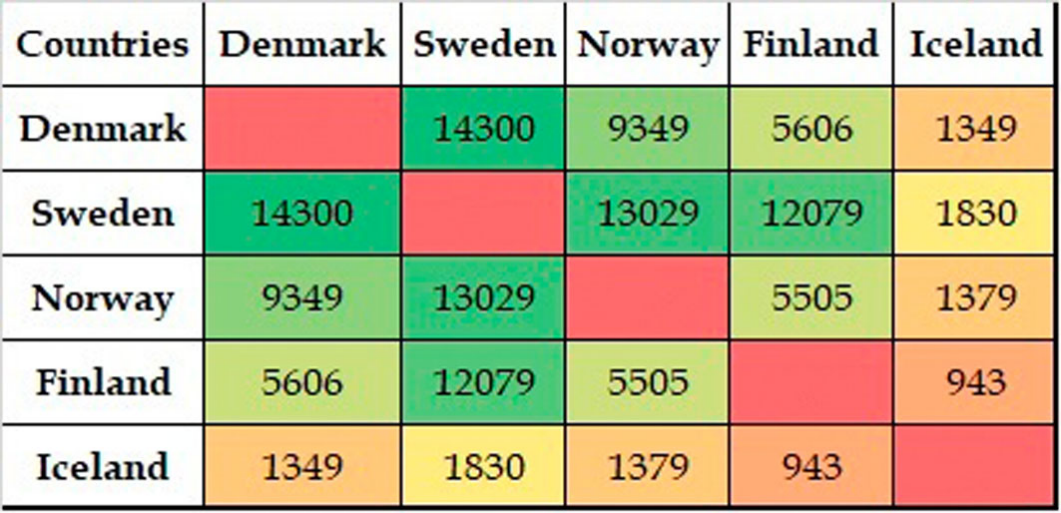
Publication distribution among Nordic countries.

In the previous sections, we have seen the collaborative outcomes of researchers in Nordic countries with researchers in other parts of the world and with industrial personnel. This section presents an analysis of the publication behavior of Nordic countries. Figure 6 shows the collaborative activities among Nordic countries. Data from smaller Nordic countries such as the Faroe Islands and Svalbard are also included. In the figure, the complete circle represents the total publications of the Nordic countries. The circle is divided into segments based on the numbers contributed by each country. Each segment is connected to the other segments using line segments. The thickness of these lines represents the number of publications. Figure 7 shows the figures for collaborative publications among Nordic countries. Denmark has the largest number of collaborative publications with Sweden, followed by Norway, Finland, and Iceland. Sweden has characteristics similar to those of Denmark, insofar as the collaboration with Denmark is highest, followed by Norway, Finland, and Iceland. Norway shows a slightly different behavior in which the highest numbers for collaborative publications are with Sweden, followed by Denmark, Finland, and Iceland. Finland has its largest number of collaborative publications with Sweden, Denmark, and Norway, with similar figures in terms of collaborations, followed by collaborations with Iceland. The highest number of collaborative publications in Iceland is with Sweden, followed by Norway, Denmark, and Finland.

**Figure 8.**
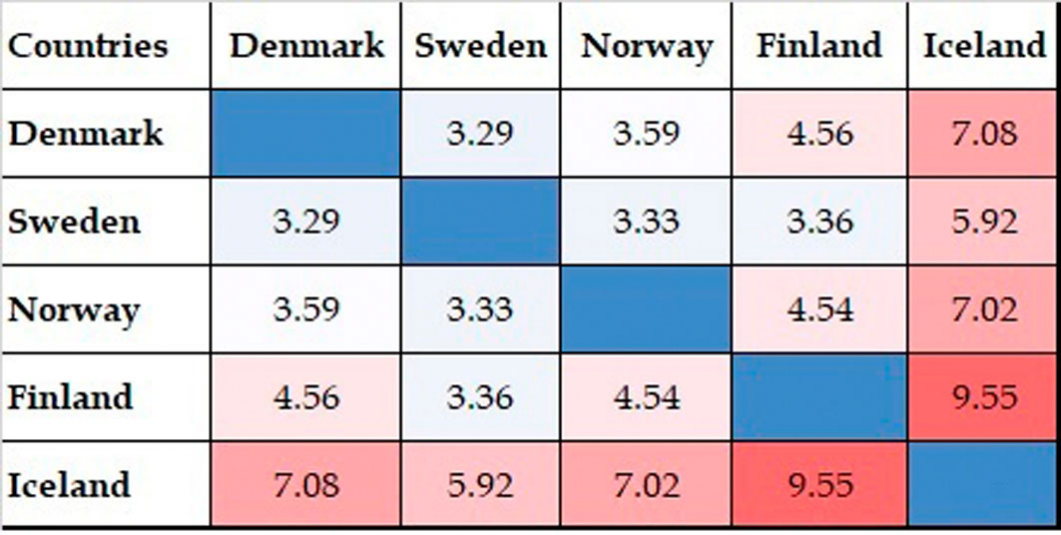
FWCI of collaborative publications among Nordic countries.

Figure 8 shows the results of the qualitative analysis of collaboration outcomes in terms of citations, as shown in the FWCI for collaborative publications. FWCI trends differ slightly from the numbers of published collaborative papers. Collaborative publications with Iceland have a higher average FWCI than any other collaboration among Nordic countries. This rate is followed by the figures for collaborative publications with Finland, Norway, Denmark, and Sweden. Collaborative publications have a higher FWCI than noncollaborative publications. Collaborative publications with Nordic countries also have a comparatively better FWCI than those with other parts of the world.

### Publications in top Quartile

**Figure 9.**
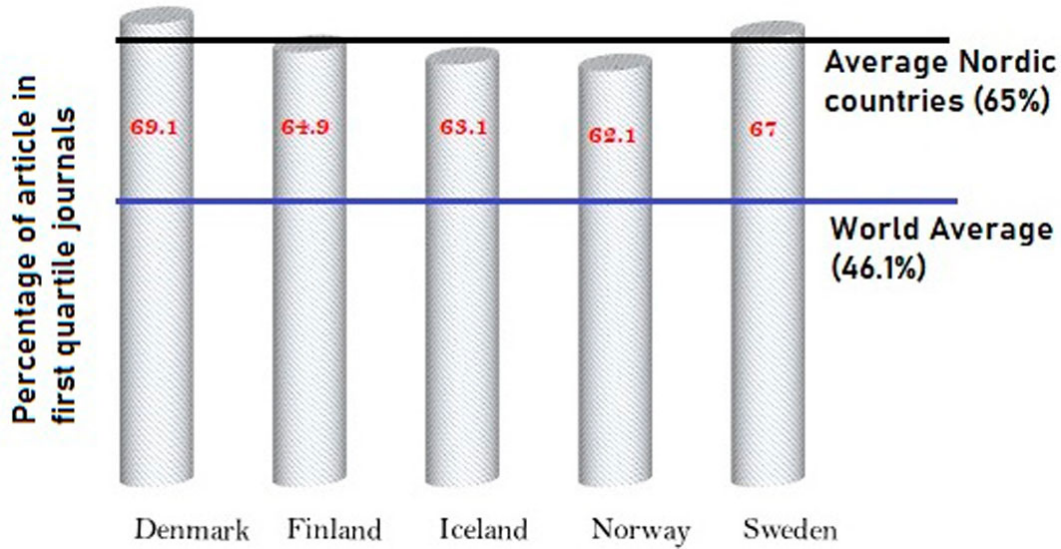
Publication in top quartile journals.

The quality of journals considered for publishing is one of the parameters indicative of the quality of research. Publication in top-rated journals signifies the quality of research. Figure 9 shows the output of Nordic countries in the top 25 percentage journals in their respective subject areas. Across the globe, overall, 46.1% of the papers published are in quartile one journals. However, in Nordic countries, approximately 65% of the papers published are in top quartile journals. This is a clear indication that the work produced by Nordic countries is of higher quality when compared to global trends. From the Nordic countries, approximately 69% of papers published in Denmark are in top quartile journals, followed by Sweden, with 67% of its publication in top quartile journals. Finland publishes 64.9% of its research studies in top quartile journals; 63.1% of publications from Iceland are in top quartile journals; and 62.1% of publications from Norway are in top quartile journals. This analysis clearly indicates that Nordic countries publish in high-quality journals, and because of this, the citation indexes are higher compared to global averages.

### Classification of publication across different subject areas by Nordic countries

**Figure 10.**
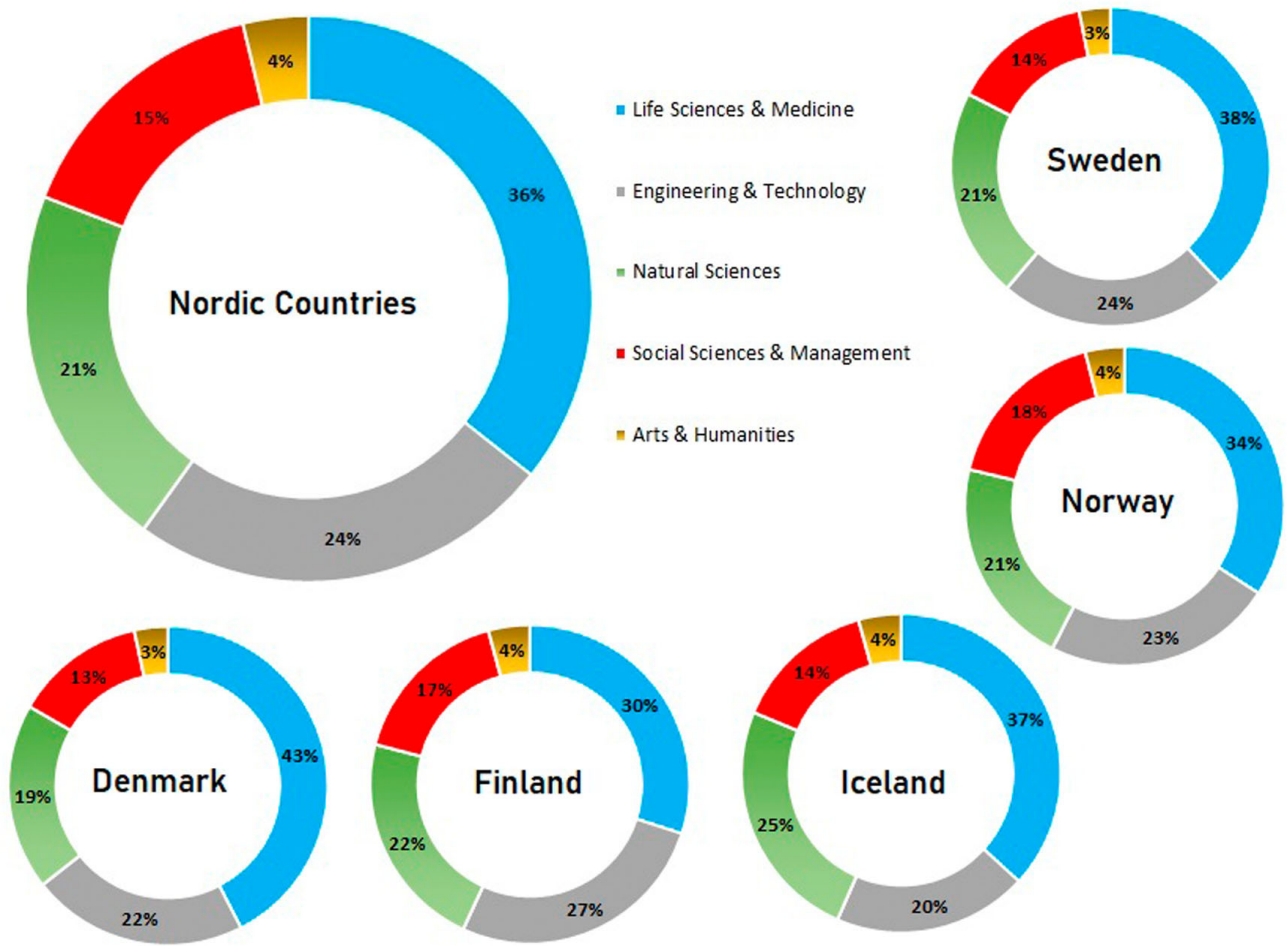
Breakdown of publications across subjects in various countries.

Journals are classified into different categories based on the specialization in which the journal publishes articles. Each journal publishes articles from a specific field. The subject areas are classified based on the standard All Journal Science Code (AJSC). All journals are associated with one or more AJSC codes. Multidisciplinary journals are associated with more than one subject area. In the present research, an attempt is made to understand the pattern of publication from Nordic countries across different subject areas. There are approximately 250+ subcategories in the AJSC. In the current study, we consider five basic subject areas: life sciences and medicine, engineering and technology, natural sciences, social sciences and management, and arts and humanities. Figure 10 shows the distribution of publications across Nordic countries as a whole and in the five individual countries. In Nordic countries, publications in life sciences and medicine are highest at 36% of overall publications, followed by engineering and technology at 24% of overall publication numbers. Publications in natural sciences are at 21%, social sciences and management are at 15%, and publications in arts and humanities constitute approximately 4% of overall numbers.

Figure 10 also shows the publication trends of individual Nordic countries in various subject areas. In all Nordic countries, publications in the area of life and medicine are highest, with 38% in Sweden, 34% in Norway, 37% in Iceland, 30% in Finland, and 43% in Denmark. This trend is followed across Nordic countries as a whole. Publications in the area of engineering and technology are the second largest, after life sciences and medicine in Sweden (24%), Norway (23%), Finland (27%), and Denmark (20%). However, the trend in Iceland is a little different, whereby publication numbers in natural sciences are second highest at 25% and those in engineering and technology are 20%. The third highest publication numbers in other Nordic countries are in natural sciences, with Sweden at 21%, Norway at 21%, Finland at 22%, and Denmark at 19%. Publications in social sciences and management are fourth highest in all Nordic countries, with 14% in Sweden, 18% in Norway, 14% in Iceland, 17% in Finland, and 13% in Denmark. The remaining number of publications are in the area of arts and humanities across all the Nordic countries. The trends are similar, with 3% in Sweden and Denmark and 4% in Norway, Finland, and Iceland.

**Figure 11.**
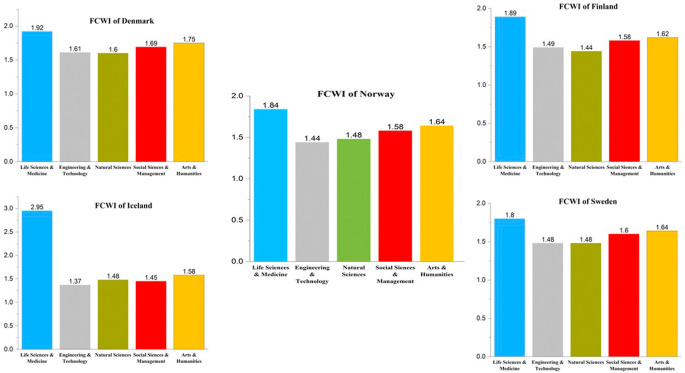
Field weighted citation index of publications in subject areas across Nordic countries.

Figure 11 presents a detailed analysis with respect to citations across different subject areas in Nordic countries. The FWCI of publications from 2016 to 2021 is presented across different subject areas for the Nordic countries. As with publication numbers, the citation impact trends are similar across Nordic countries. Publications in life sciences and medicine have the highest impact score compared to all other subject areas in all the Nordic countries, with Norway at 1.84, Sweden at 1.8, Finland at 1.89, Iceland at 2.95, and Denmark at 1.92. Of all the Nordic countries, Iceland has the highest quality factor. This could be due to the large percentage of collaborative work. The second highest citation index is not like the publication numbers. The subject for which Norway has the smallest publication number (arts and humanities) has an FWCI at 1.64; Sweden is at 1.64; Finland is at 1.62; Iceland is at 1.58; and Denmark is at 1.75. The behavior of the citation index in the remainder of the subject area across Nordic countries is not uniform, as is the case with the first or second position elements. The FWCI in the area of engineering and technology is 1.44 in Norway, 1.48 in Sweden, 1.49 in Finland, 1.37 in Iceland and 1.61 in Denmark. The FWCI for natural sciences in Nordic countries is 1.48 in Norway, 1.48 in Sweden, 1.44 in Finland, 1.48 in Iceland, and 1.6 in Denmark. Finally, the FWCI in social sciences and management is 1.58 in Norway, 1.6 in Sweden, 1.58 in Finland, 1.48 in Iceland, and 1.69 in Denmark.

## Conclusion

The current study was done to analyse the research outcomes of Nordic countries using Scopus, Scival and World Bank Data. The research population and research spent of different countries was compared. Among Nordic countries, Sweden, with the highest population, also has the highest researcher population. This analysis has shown that in this region, research spent and the number of researchers have shown a positive correlation with the FWCI, a citation metric that considers the differences in research behavior across different disciplines. Sweden, with the highest number of researchers, also has the highest proportion of GDP spent on research among the Nordic countries.

However, Iceland, with a low research expenditure, has a high FWCI. On studying the collaboration within the Nordic countries, it was observed that Sweden and Denmark have the highest number of coauthored publications. Within Nordic countries, the highest FWCI is seen in coauthored publications of Iceland and Finland, closely followed by Iceland and Denmark and finally Iceland and Norway. Iceland thus shows the highest FWCI within Nordic countries despite having the smallest researcher population. All the countries in this region have a high number of coauthored publications with the United States. The only exception, again, is Iceland, which has the highest number of coauthored publications with the US, followed by Sweden. The distribution of coauthored publications across continents shows that all Nordic countries have the highest numbers of collaborations within Europe. Denmark has the highest percentage of publications in collaboration with the corporate sector, which is followed by Iceland. Denmark tops the Nordic countries with the highest number of publications in first quartile journals while Sweden ranks second. This implies that Denmark tops the list in quality publications. Life Sciences and medicine, Engineering and Technology and Natural Sciences are the top three areas in which Nordic countries are contributing. On comparing research across different subject areas, all countries have the highest number of publications in life sciences and medicine. There is also a significant contribution by Nordic countries in social sciences and management and arts and humanities. Overall, Nordic countries produce high numbers of publications across different subject areas and, in addition to global collaborations, have a large chunk of researchers who collaborate with different European nations. Having the FWCI of all the countries above the world average makes the research outcomes of this region promising. Due to the proximity of these nations and the common challenges encountered, Nordic countries, with their versatile potential in different areas of research, can work as a team to build a strong multidisciplinary research hub. This study points towards the finding that collaborative research can help in increasing the quality and thus research citations as shown in Nordic countries.

This study puts forth the research outcomes and research strengths of Nordic countries. Such information can help the researchers in different areas globally to collaborate with the researchers in these nations. The clarity about the research strength of these nations can help funders to make strategies for funding these nations. Also, this information can help other countries to follow various practices to improve the research impact.

## Data Availability

Open Science Framework: Extended data for ‘Bibliometric analysis of Nordic countries for the period 2016 to 2020’ is archived at https://doi.org/10.17605/OSF.IO/7MAZ4. ^46^ This project contains the following underlying data:
• data_file.xlsx – Data related to bibliographic data of Nordic countries with respect to subjects. • working.xlsx – Data related to ranking and world bank parameter of Nordic countries Data are available under the terms of the Creative Commons Zero “No rights reserved” data waiver (CC0 1.0 Public domain dedication).

## References

[1] Kautto M : The nordic countries. *The Oxford handbook of the welfare state.* 2010.

[2] Eskildsen JK Kristensen K Westlund AH : Work motivation and job satisfaction in the Nordic countries. *Empl. Relat.* 2004;26:122–136. 10.1108/01425450410511043

[3] Lane JE Ersson S : The Nordic Countries. *Political institutions in Europe.* 2002;245.

[4] Kangas O Palme J : Social policy and economic development in the Nordic countries: an introduction. *Social policy and economic development in the Nordic countries.* London: Palgrave Macmillan;2005; (pp.1–16).

[5] Martela F Greve B Rothstein B : The Nordic exceptionalism: what explains why the Nordic Countries are constantly among the happiest in the world. Helliwell JF Layard R Sachs JD editors. *World Happiness Report.* 2020; pp.128–145.

[6] Furu K Wettermark B Andersen M : The Nordic countries as a cohort for pharmacoepidemiological research. *Basic Clin. Pharmacol. Toxicol.* 2010;106(2):86–94. 10.1111/j.1742-7843.2009.00494.x 19961477

[7] Lin CYY Edvinsson L : National intellectual capital: comparison of the Nordic countries. *J. Intellect. Cap.* 2008.

[8] Michelsen S Stenström ML Jørgensen CH : *Vocational education in the nordic countries.* Routledge;2018.

[9] Tøssebro J Bonfils IS Teittinen A : Normalization fifty years beyond—current trends in the Nordic countries. *J. Policy Pract. Intellect. Disabil.* 2012;9(2):134–146. 10.1111/j.1741-1130.2012.00340.x

[10] Lööf H Heshmati A Asplund R : *Innovation and performance in manufacturing industries: A comparison of the Nordic countries*(No. 457). SSE/EFI working paper series in economics and finance.2001.

[11] Glänzel W : Science in Scandinavia: A bibliometric approach. *Scientometrics.* 2000;48(2):121–150. 10.1023/A:1005640604267

[12] Clarke A Gatineau M Grimaud O : A bibliometric overview of public health research in Europe. *Eur. J. Pub. Health.* 2007;17(suppl_1):43–49. 10.1093/eurpub/ckm063 17666422

[13] Melin G Persson O : Hotel cosmopolitan: A bibliometric study of collaboration at some European universities. *J. Am. Soc. Inf. Sci.* 1998;49(1):43–48. 10.1002/(SICI)1097-4571(1998)49:1<43::AID-ASI6>3.0.CO;2-R

[14] Gjersvik P Nylenna M Jemec GB : Dermatologic research in the Nordic countries 1989–2008–a bibliometric study. *Int. J. Dermatol.* 2010;49(11):1276–1281. 10.1111/j.1365-4632.2010.04508.x 21038549

[15] Schneider JW : Bibliometric Research Performance Indicators for the Nordic Countries: A publication from the NORIA-net “The use of bibliometrics in research policy and evaluation activities”. 2010.

[16] Sandnes FE : A bibliometric study of human–computer interaction research activity in the Nordic-Baltic Eight countries. *Scientometrics.* 2021;126(6):4733–4767. 10.1007/s11192-021-03940-z

[17] Glänzel W Schlemmer B Schubert A : Proceedings literature as additional data source for bibliometric analysis. *Scientometrics.* 2006;68(3):457–473. 10.1007/s11192-006-0124-y

[18] Koskinen J Isohanni M Paajala H : How to use bibliometric methods in evaluation of scientific research? An example from Finnish schizophrenia research. *Nord. J. Psychiatry.* 2008;62(2):136–143. 10.1080/08039480801961667 18569777

[19] Tarkowski SM : Environmental health research in Europe–bibliometric analysis. *Eur. J. Pub. Health.* 2007;17(suppl_1):14–18. 10.1093/eurpub/ckm065 17666416

[20] Rabow H : Bibliometric research output indicators and university funding in the Nordic countries. *ScieCom Info.* 2012;8(1).

[21] Merigó JM Gil-Lafuente AM Yager RR : An overview of fuzzy research with bibliometric indicators. *Appl. Soft Comput.* 2015;27:420–433. 10.1016/j.asoc.2014.10.035

[22] Hanvold TN Kines P Nykänen M : Occupational safety and health among young workers in the Nordic countries: a systematic literature review. *Saf. Health Work.* 2019;10(1):3–20. 10.1016/j.shaw.2018.12.003 30949376 PMC6429009

[23] Widfeldt A : The radical right in the Nordic Countries. *The Oxford handbook of the radical right.* 2018.

[24] Knudsen JP : Dealing with rural–urban economic welfare challenges in the Nordic countries–a theory-based overview. *Nordisk välfärdsforskning|Nordic Welfare Research.* 2020;5(1):58–69. 10.18261/issn.2464-4161-2020-01-06

[25] Thomsen JP Bertilsson E Dalberg T : Higher education participation in the Nordic countries 1985–2010—a comparative perspective. *Eur. Sociol. Rev.* 2017;33(1):98–111.

[26] Airey J Lauridsen KM Räsänen A : The expansion of English-medium instruction in the Nordic countries: Can top-down university language policies encourage bottom-up disciplinary literacy goals? *High. Educ.* 2017;73(4):561–576. 10.1007/s10734-015-9950-2

[27] Skogerbø E Ihlen Ø Nörgaard Kristensen N : Power, communication, and politics in the Nordic countries. Nordicom. 2021.

[28] Christensen S Zhang W : Shadow Education in the Nordic Countries: An Emerging Phenomenon in Comparative Perspective. *ECNU Rev. Educ.* 2021;4(3):431–441. 10.1177/20965311211037925

[29] Tikkanen T : Problem-solving skills, skills needs and participation in lifelong learning in technology-intensive work in the Nordic countries. *Journal of Contemporary Educational Studies/Sodobna Pedagogika.* 2017;68(4).

[30] Baccini A De Nicolao G Petrovich E : Citation gaming induced by bibliometric evaluation: A country-level comparative analysis. *PLoS One.* 2019;14(9):e0221212. 10.1371/journal.pone.0221212 31509555 PMC6739054

[31] López-Illescas C Moya Anegón F de Moed HF : Comparing bibliometric country-by-country rankings derived from the Web of Science and Scopus: The effect of poorly cited journals in oncology. *J. Inf. Sci.* 2009;35(2):244–256. 10.1177/0165551508098603

[32] Zacca-González G Chinchilla-Rodríguez Z Vargas-Quesada B : Bibliometric analysis of regional Latin America’s scientific output in public health through SCImago journal & country rank. *BMC Public Health.* 2014;14(1):1–11. 10.1186/1471-2458-14-632 24950735 PMC4094685

[33] Khiste GP Paithankar RR : Analysis of Bibliometric term in Scopus. *International Journal of Library Science and Information Management (IJLSIM).* 2017;3(3):81–88.

[34] Tibaná-Herrera G Fernández-Bajón MT Moya Anegón F de : Output, collaboration and impact of e-learning research: Bibliometric analysis and visualizations at the country and institutional level (Scopus 2003-2016). *Profesional de la Información.* 2018;27:1082. 10.3145/epi.2018.sep.12

[35] Shehatta I Al-Rubaish AM : Impact of country self-citations on bibliometric indicators and ranking of most productive countries. *Scientometrics.* 2019;120(2):775–791. 10.1007/s11192-019-03139-3

[36] Hassan-Montero Y Guerrero-Bote VP De-Moya-Anegón F : Graphical interface of the Scimago Journal and Country Rank: an interactive approach to accessing bibliometric information. *El profesional de la información.* 2014;23(3):272–278. 10.3145/epi.2014.may.07

[37] Archambault É Campbell D Gingras Y : Comparing bibliometric statistics obtained from the Web of Science and Scopus. *J. Am. Soc. Inf. Sci. Technol.* 2009;60(7):1320–1326. 10.1002/asi.21062

[38] Lin G Hu Z Hou H : Research preferences of the G20 countries: a bibliometrics and visualization analysis. *Curr. Sci. (00113891).* 2018;115(8):1477. 10.18520/cs/v115/i8/1477-1485

[39] Corrall S Kennan MA Afzal W : Bibliometrics and research data management services: Emerging trends in library support for research. *Libr. Trends.* 2013;61(3):636–674. 10.1353/lib.2013.0005

[40] Miremadi I Saboohi Y Arasti M : The influence of public R&D and knowledge spillovers on the development of renewable energy sources: The case of the Nordic countries. *Technol. Forecast. Soc. Chang.* 2019;146:450–463. 10.1016/j.techfore.2019.04.020

[41] De la Porte C Jensen MD Kvist J : Going Nordic—Can the Nordic model tackle grand challenges and be a beacon to follow? *Regul. Gov.* 2023;17(3):595–607. 10.1111/rego.12494

[42] Karseth B Sivesind K Steiner-Khamsi G : *Evidence and expertise in Nordic education policy: A comparative network analysis.* Springer Nature;2022; p.429. 10.1007/978-3-030-91959-7

[43] Verger A : Evidence-Based Policy Making and Educational Reform in Nordic Europe: Key Contributions of the POLNET Study. *Evidence and Expertise in Nordic Education Policy: A Comparative Network Analysis.* Cham: Springer International Publishing;2022; pp.395–408. 10.1007/978-3-030-91959-7_14

[44] Andersson C Sund K : Technical Efficiency and Productivity of Higher Education Institutions in the Nordic Countries. *Int. J. Public Adm.* 2022;45(2):107–120. 10.1080/01900692.2020.1868508

[45] Sivesind K Karseth B : Introduction: A Comparative Network Analysis of Knowledge Use in Nordic Education Policies. *Evidence and Expertise in Nordic Education Policy: A Comparative Network Analysis.* Cham: Springer International Publishing;2022; pp.1–31. 10.1007/978-3-030-91959-7_1

[46] Santhosh KV Janodia M Magazine BC : Nordic countries[Dataset.]2023, April 29. 10.17605/OSF.IO/7MAZ4

