# Supplementary material for: Mapping the Nordic Research Landscape for the period 2016-2020: a comprehensive study of research outcomes, collaborations, and impact
Source: F1000Res. 2024 Jul 29;13:71. Originally published 2024 Jan 15. [Version 2] doi: 10.12688/f1000research.144036.2 (PMC11387935; doi:10.12688/f1000research.144036.2)
Supplement: Peer review history

# Reviewer report: Reviewer response for version 2

**DOI:** 10.5256/f1000research.169327.r315892

**Published:** 2024-10-18

**Authors:** Zhen Yang

**Affiliations:** 1 KU Leuven, Leuven, Flanders, Belgium

Many thanks to the editors for inviting me to review this interesting manuscript, and my suggestions are as follows:

 Comment 1:

 Methods:

 Can you explain why the Scopus database was used instead of other large multidisciplinary databases (e.g. Web of Science)?

 Comment 2:

 Methods:

 The use of a single database in bibliometrics inevitably leaves some studies missing, so why not merge multiple databases?

 Comment 3:

 Methods:

 The date of inclusion in the literature could be more detailed, e.g. 1 January 2016. In addition, why was data extracted over two days rather than a shorter period? Large electronic databases are updated very frequently and extracting data on different calendar days could lead to discrepancies.

 Comment 4:

 I think it would be useful to include a discussion of the limitations of this study, particularly the bibliometric methodological limitations.

Is the work clearly and accurately presented and does it cite the current literature?

Yes

If applicable, is the statistical analysis and its interpretation appropriate?

Partly

Are all the source data underlying the results available to ensure full reproducibility?

Yes

Is the study design appropriate and is the work technically sound?

Partly

Are the conclusions drawn adequately supported by the results?

Yes

Are sufficient details of methods and analysis provided to allow replication by others?

Partly

Reviewer Expertise:

Physical activity for health, bibliometric analysis

I confirm that I have read this submission and believe that I have an appropriate level of expertise to confirm that it is of an acceptable scientific standard, however I have significant reservations, as outlined above.

# Reviewer report: Reviewer response for version 2

**DOI:** 10.5256/f1000research.169327.r308398

**Published:** 2024-09-10

**Authors:** Palanisamy Sivanandy

**Affiliations:** 1 Department of Pharmacy Practice, International Medical University, Kuala Lumpur, Malaysia

Approved

Is the work clearly and accurately presented and does it cite the current literature?

Partly

If applicable, is the statistical analysis and its interpretation appropriate?

Partly

Are all the source data underlying the results available to ensure full reproducibility?

Yes

Is the study design appropriate and is the work technically sound?

Yes

Are the conclusions drawn adequately supported by the results?

Yes

Are sufficient details of methods and analysis provided to allow replication by others?

Partly

Reviewer Expertise:

Medical Science, Health and Life Sciences

I confirm that I have read this submission and believe that I have an appropriate level of expertise to confirm that it is of an acceptable scientific standard.

# Reviewer report: Reviewer response for version 1

**DOI:** 10.5256/f1000research.157773.r297294

**Published:** 2024-07-10

**Authors:** Palanisamy Sivanandy

**Affiliations:** 1 Department of Pharmacy Practice, International Medical University, Kuala Lumpur, Malaysia

1. What is the rationale for comparing the country's population and the number of researchers? any significant findings using these values? must be explained in the abstract and discussion section.

 2. It is good to explain the data extraction using a structured flowchart with the number of articles from every source like PubMed, Science Direct, Cochrane, etc.

 3. The statistical tests applied in this research are not included fully. In the method section, only MS Excel was mentioned, however, it is better to add a correlation analysis of publications with different countries and impact factor/citation indexes. 

 4. Figure 3, compares the research spending by percentage of GDP among the Nordic countries." How GDP was compared with the research spending? need to be explained in the method/results section.

 5. Why data from the Faroe Islands and Greenland were excluded? Explain the reasons. 

 6. Throughout the article it was mentioned as more publications on life sciences. Life science is a broad terminology, it is better to segregate the articles within the subsection/class of life sciences.

Is the work clearly and accurately presented and does it cite the current literature?

Partly

If applicable, is the statistical analysis and its interpretation appropriate?

Partly

Are all the source data underlying the results available to ensure full reproducibility?

Yes

Is the study design appropriate and is the work technically sound?

Yes

Are the conclusions drawn adequately supported by the results?

Yes

Are sufficient details of methods and analysis provided to allow replication by others?

Partly

Reviewer Expertise:

Medical Science, Health and Life Sciences

I confirm that I have read this submission and believe that I have an appropriate level of expertise to confirm that it is of an acceptable scientific standard, however I have significant reservations, as outlined above.

# Response

**Published:** 2024-07-31

**Authors:** Santhosh Venkata

**Affiliations:** Manipal Academy of Higher Education, India

What is the rationale for comparing the country's population and the number of researchers? any significant findings using these values? must be explained in the abstract and discussion section.

**Response**: There is an uneven distribution of researchers in the world. By comparing, the number of researchers with population gives an idea about the percent growth of researcher’s pool when the country’s population is increasing. We attempted to understand researcher per 100000 population in these countries and its correlation with the % of GDP spend on research. Hence, we compared population with researchers. The rationale is explained in paragraph above fig 2.

 It is good to explain the data extraction using a structured flowchart with the number of articles from every source like PubMed, Science Direct, Cochrane, etc.

**Response**: Since this is a bibliometric study, the data was extracted from databases like Scopus and SciVal, which are products from Elsevier. For this study, we did not use PubMed, ScienceDirect or Cochrane as bibliometric data cannot be accessed from these databases.

 The statistical tests applied in this research are not included fully. In the method section, only MS Excel was mentioned, however, it is better to add a correlation analysis of publications with different countries and impact factor/citation indexes.

**Response**: With respect to the objectives of the study, there are no statistical tests applied. The bibliometric data from Scopus and SciVal was extracted in the form of excel sheet for analysis. There is no correlation studied in this manuscript. The impact factor as a metric, is used for journals and not at the country level. In the bibliometric studies the accepted citation index is FWCI, which we have mentioned in the manuscript. However, added Correlation of Countries with bibliographic metrics (Scholarly Output, Citation, Citation per Publication, FWCI) and Correlation of co-authored publication among countries

 Figure 3, compares the research spending by percentage of GDP among the Nordic countries." How was GDP compared with the research spending? need to be explained in the method/results section.

**Response**: As per the World Bank Data, the research spent is considered as percentage of GDP. So we have shown in figure 3 the relation of research spent with respect to FWCI.  Research spent percentage gives a fair idea of ruling governments commitment to research growth 

 Why data from the Faroe Islands and Greenland were excluded? Explain the reasons.

**Response:** Most literature on Nordic countries emphasize on prominent five countries studied. Further, Faroe Islands is an autonomous region governed by Denmark, which is already studied. Moreover, these are small islands with maximum of 60,000 population with one university in each of the islands.  Also, the population in this area is predominantly migratory and thus this area will not give a correct dynamic of research productivity in alignment with other countries.

 Throughout the article it was mentioned as more publications on life sciences. Life science is a broad terminology, it is better to segregate the articles within the subsection/class of life sciences.

**Response**: The term “Life Science” is a category identified by the Scopus and SciVal. Since, this is a broad term used to describe in Scopus, along with other broad domains based on ranking agencies (five broad categories for the QS and 11 categories for THE), we have refrained from further subclassification of the term life science.

# Reviewer report: Reviewer response for version 1

**DOI:** 10.5256/f1000research.157773.r276617

**Published:** 2024-06-08

**Authors:** Mats Benner

**Affiliations:** 1 Lunds universitet, Lund, Sweden

The paper affords a descriptive overview of research conditions in the Nordic countries, citing the relevant sources and providing information in an attainable and accessible form. What it does not do is to afford an analysis of the Nordic countries beyond that, i.e. how the composition of the research systems have evolved time time, why collaborative patterns take the form they take, and what overall conclusions can be drawn. There is an abundance of literature on the evolution of research system, how funding arrangements are aligned with over policy priorities, and forms of implementation at the level of universities, companies and institutes, and similar. Some of the literature deal with overall steering principles for research (i.e. principal-agent, systems of innovation, governance models, etc), others with the historical and comparative development of institutional arrangements and the relationship between inputs (funding primarily) and outputs (publications, citations, patents). The text does not engage with these literatures. What it does is to present an overall descriptive account of the state of research in the Nordics based on openly available statistics. This means that its contributions are exclusively at the level of description, not theory or analysis.

Is the work clearly and accurately presented and does it cite the current literature?

Yes

If applicable, is the statistical analysis and its interpretation appropriate?

Yes

Are all the source data underlying the results available to ensure full reproducibility?

Yes

Is the study design appropriate and is the work technically sound?

Yes

Are the conclusions drawn adequately supported by the results?

Yes

Are sufficient details of methods and analysis provided to allow replication by others?

Yes

Reviewer Expertise:

Research policy

I confirm that I have read this submission and believe that I have an appropriate level of expertise to confirm that it is of an acceptable scientific standard.
